# Blood test shows high accuracy in detecting stage I non-small cell lung cancer

**DOI:** 10.1186/s12885-020-6625-x

**Published:** 2020-02-21

**Authors:** Cherylle Goebel, Christopher L. Louden, Robert Mckenna, Osita Onugha, Andrew Wachtel, Thomas Long

**Affiliations:** 1 Goebel Consulting Inc., Mountain View, 780 Montague Expressway, Suite 703, San Jose, CA 95131 USA; 2 Louden Consulting, San Antonio, TX USA; 3 Providence Saint John’s Health Center/John Wayne Cancer Institute, Santa Monica, CA USA; 4 Southern California Institute for Respiratory Diseases, Los Angeles, CA USA; 5 Lung Cancer Proteomics LLC, Hebron, IN USA

**Keywords:** Immunoassay, Early stage lung cancer, Detection, Biomarkers, Proteomics, Diagnostic tests, Non-small cell lung cancer

## Abstract

**Background:**

In a previous study (Goebel et. al, Cancer Genomics Proteomics 16:229-244, 2019), we identified 33 biomarkers for an early stage (I-II) Non-Small Cell Lung Cancer (NSCLC) test with 90% accuracy, 80.3% sensitivity, and 95.4% specificity. For the current study, we used a narrowed ensemble of 21 biomarkers while retaining similar accuracy in detecting early stage lung cancer.

**Methods:**

A multiplex platform, 486 human plasma samples, and 21 biomarkers were used to develop and validate our algorithm which detects early stage NSCLC. The training set consisted of 258 human plasma with 79 Stage I-II NSCLC samples. The 21 biomarkers with the statistical model (Lung Cancer Detector Test 1, LCDT1) was then validated using 228 novel samples which included 55 Stage I NSCLC.

**Results:**

The LCDT1 exhibited 95.6% accuracy, 89.1% sensitivity, and 97.7% specificity in detecting Stage I NSCLC on the blind set. When only NSCLC cancers were analyzed, the specificity increased to 99.1%.

**Conclusions:**

Compared to current approved clinical methods for diagnosing NSCLC, the LCDT1 greatly improves accuracy while being non-invasive; a simple, cost-effective, early diagnostic blood test should result in expanding access and increase survival rate.

## Background

Lung cancer is a pervasive disease that is commonly diagnosed at a late stage and has a global estimated mortality rate of 84.2% for 2019. The American Cancer Society predicts about 228,150 new cases and 142,670 deaths for 2019 in the United States. On average, 422 Americans die every day of lung cancer (LC). Non-small Cell Lung Cancer (NSCLC) accounts for 84% of all LCs, and exhibits a 5-year survival rate of 23% [1]. However, if NSCLC is detected at stages I-II, the survival rate significantly improves and it may even be cured [2]. NSCLC patients diagnosed at stage I have a 5-year survival rate between 68 and 92%; at stage II between 53 and 60%; at stage III, it decreases to 26–36%; and for stage IV detection the survival rate drops precipitously to 1–10% [1, 2]. Despite significant investment and advancement in LC research, only 16% of LCs are detected at the early stages [1].

The research community continues to improve early LC detection through the use of Computed Tomography (CT) and Positron Emission Tomography (PET) scans, improved pulmonary nodule (PN) guideline, biomarkers, and machine learning algorithms. In our approach, we combine biomarkers and machine learning. We detect early stage NSCLC with high sensitivity and specificity using a simple blood test. Before presenting our results, we briefly review the state-of-the-art of these approaches.

### CT scans recommended for diagnosing LC

The US Preventive Service Task Force (USPSTF) recommends that low-dose computed tomography (LDCT) scans be used as a screening method for LC in high risk patients age 55–85 with a 30-year smoking history, who have not quit for more than 15 years. The recommendation was in part based on the National Lung Screening Trial (NLST) study which demonstrated that screening with LDCT reduces LC mortality by 20% compared to chest x-rays [3, 4]. However, this approach is not ideal.

In the NLST study, a PN was detected in 1 of every 4 subjects that had LDCT scans. Of the 7191 subjects found to have suspicious nodules on LDCT scans, 88.6% had a follow-up test (e.g.*,* imaging, 89.8%; biopsy, 1.9%; and surgery, 4.7%), and only 292 (4.1%) were confirmed to have LC. Of these 292 cases, 54.1 and 41.1%, turned out to be Stage I and II, respectively. The LDCT scans had a false positive rate (FPR) of 96.1% [4]. Obviously, there is a need for a test with a lower FPR. Deep learning algorithms show promise to reduce the false positives in interpreting these images [5].

### PET scans increasing in use for LC follow-ups

PET scans have better statistics than LDCT [6]. A multi-center observational study by Tanner, et al., [7] evaluating PN management shows an increase in PET scan use with additional follow-ups of patients with indeterminate lung nodules. The accuracy rate of PET scans is 74%, with an overall FPR of 39% (36–55%) and overall false negative rate (FNR) of 9% (8–10%), depending on node size. The study concludes that 25% of PNs referred to a pulmonologist were malignant; 46% had additional surveillance, 33.2% had a biopsy, and 20.4% underwent lung surgery. About 35% of patients who had surgery had benign masses.

### Pulmonary nodule guidelines

Most solitary PNs are detected incidentally by chest radiography and CT scans that were ordered to investigate other diseases. Approximately 150,000 solitary PNs are detected annually in the United States of America [8].

Recommendations for managing intermediate PNs, found in PET/CTs using the Lung-RADS [7, 9] or Fleischner criteria [10], are not always followed. Many physicians consider other factors, such as age, smoking status, gender, patient preference, and use their experience when deciding on follow-up procedures for that patient’s specific clinical situation. In a multicenter observational study of 377 patients, Tanner et al., indicated that invasive procedures were performed in 44% of low risk nodules (< 5% probability of malignancy) [7]. Today, current guidelines for management of lung nodules try to incorporate other factors that may be unique to a patient [9, 10]. Prospective research on physician adherence to new guidelines and outcome on performed PN follow-up procedures will need to be completed.

### Evaluating biomarkers to detect LC

There is a growing trend to use genetic and protein biomarkers for disease diagnosis, prognosis, and the evaluation of treatment efficacy (e.g., Grail, Guardant, Myriad Genetics) [11, 12]. Biomarkers are defined as ‘*any substance, structure, or process that can be measured in the body or its products and influence or predict outcome or disease*’ [13]. Thus, a biomarker can be of physical, chemical, or biological nature, such as measurements of blood pressure, temperature, inflammatory cytokines (proteins), genetic (DNA) markers, or metabolites [14]. In this paper, we will limit our discussion to DNA and protein biomarkers.

#### DNA biomarkers

DNA biomarkers have been used to assess risk for developing specific diseases or response to therapy. DNA provides genetic information of the individual. Nonetheless, the path from DNA to an observable physical trait (e.g., disease) is complex. For instance, somatic mutations in the TP53, EGFR, and KRAS genes are commonly found in LC patients [15]; yet, somatic mutations are often due to increased exposure to carcinogens (e.g., smoking, radon), environmental factors (e.g., pollution, second-hand smoke), age, and health history (e.g., chronic COPD). Inherited mutations following an autosomal dominant pattern predispose an individual to be at high risk, but need not always predict the development of LC. The pattern of inheritance, penetrance, and expressivity of genetic mutations, in addition to lifestyle, environmental factors, and even ethnicity, are important components in assessing cancer risk [16].

#### Protein biomarkers

In contrast, protein reflects phenotype: the observable end-trait (e.g., tissue) resulting from the interaction of genome and the environment [17]. Protein biomarkers provide quantitative data that can be compared between a healthy and a diseased individual. Proteomics has its own challenges. Proteins, like genes, are pleiotropic: meaning the same protein markers may contribute to different immune-related pathways for different diseases. For example, IL-8 is a pleiotropic cytokine and has also been linked to breast, prostate, lung, colorectal, and skin cancer [18]. Hence, using a single biomarker, *protein or DNA,* would not be sufficient for clinical diagnostic use.

Protein levels can fluctuate due to physiological stressors (e.g., disease, strenuous exercise) and samples (i.e., serum, plasma) are sensitive to environmental factors (i.e., pH, temperature) and degrade faster than DNA. Moreover, analytic protein platforms require the use of antibodies which, in turn, exhibit lot-to-lot variations due to the idiosyncratic nature of antibodies.

Despite the intricacies, genome and protein biomarkers, have proven to be essential tools in the discovery of predictive, prognostic, and diagnostic markers in LC [19–21].

### Machine learning in medicine

Advances in computing combined with an increase in the amount of data collected has enabled the application of various machine learning techniques, such as Neural Networks and Random Forests, to tease out complex and non-linear relationships in data. These methods can also assist radiologists to interpret x-rays, CAT scans, PET scans and other diagnostic imaging methods; diagnose patients with disease; and may lead to a general improvement in patient care [22].

While machine learning methods are powerful, they have drawbacks. No machine learning method can compensate for poor data (i.e., dirty data). Machine learning is unable to provide causal information on its own; they are simply a set of advanced statistical techniques that can improve our ability to find complex, non-linear relationships in data [23, 24].

Further, statistical models can be impacted by bias, human error, sample population, poor technical design, misapplication, and disparate systems. It is important that appropriate machine learning techniques and algorithms are applied to each study, that the data is collected, cleaned and processed in a consistent manner, and that bias are scrutinized from all angles [25].

Our preliminary studies identified protein biomarkers that may significantly improve our ability to identify NSCLC so this study was undertaken to prospectively test that hypothesis.

## Methods

This study is a continuation of our previous research that used 33 biomarkers [11]. Here we reduced the number of biomarkers to 21, ensured successful transfer of reagents, and retrained our algorithm.

### Study population

This study was performed on biobank plasma samples from 486 subjects distributed into 5 cohorts (Table 1). In previous studies, we demonstrated that our method detected early to late stage NSCLC. In this study, our focus was to detect stage I-II LC. Therefore, samples from patients with Stage I-II NSCLC (Table 2) were used to train the LCDT1 algorithm and, subsequently, only Stage I NSCLC samples (Table 2) were used in a blind set to validate clinical efficacy.

**Table 1:** Sample Criteria

Cohort	Inclusion	Exclusion
All cohort	M/F, 18 y/o or older, sample collected in the USA	Pregnant, incarcerated, lack of capacity to consent, samples collected outside of sthe United States
Asthma	Smoker or non-smoker	Any cancer diagnosis
Non-Smoker	Healthy	Smokers, any cancer diagnosis
NSCLC, (Stage I-II)	Stage I-II; smoker or non-smoker	Stage III-IV lung cancer
Smoker	10 pack years	Any cancer diagnosis
Other Cancers	Breast, colon-rectal, pancreatic, and prostate cancer, all stages; smoker or non-smoker	

The non-smoker and NSCLC served as negative and positive control for lung cancer, respectively. Asthma sufferer and COPDs were included to test whether the diagnostic test can differentiate lung cancer from those who may have other respiratory diseases which share similar symptoms. The smokers consisted of high-risk population for LC who were not diagnosed with any cancer. Other cancers (i.e., breast, prostate, pancreatic, and colon-rectal) were included to ensure that the diagnostic test was specific to NSCLC

**Table 2:** Sample Distribution

	African-American	Caucasian	Hispanic	Total
Training Set				
**Female**	**37**	**69**	**27**	**133**
Asthma	0	10	1	11
Breast Cancer	0	1	0	1
Colon-Rectal Cancer	0	2	0	2
Non-Smoker	15	17	10	42
NSCLC, (Stage I-II)	10	22	10	42
Pancreatic Cancer	0	2	0	2
Smoker	12	15	6	33
**Male**	**49**	**53**	**23**	**126**
Asthma	0	4	0	4
Non-Smoker	18	14	9	41
NSCLC, (Stage I-II)	9	17	10	37
Pancreatic Cancer	0	2	0	2
Smoker	22	16	4	42
**Total**	**86**	**122**	**50**	**258**
Validation Set				
**Female**	**29**	**88**	**18**	**135**
Asthma	0	8	0	8
Breast Cancer	5	35	0	40
Colon-Rectal Cancer	0	3	0	3
Non-Smoker	9	12	9	30
NSCLC, Stage I	6	17	4	27
Pancreatic Cancer	0	2	0	2
Smoker	9	11	5	25
**Male**	**25**	**51**	**17**	**93**
Asthma	0	3	0	3
Colon-Rectal Cancer	0	2	0	2
Non-Smoker	7	11	9	27
NSCLC, Stage I	5	18	5	28
Pancreatic Cancer	0	1	0	1
Prostate	3	6	0	9
Smoker	10	10	3	23
**Total**	**54**	**139**	**35**	**228**

All samples were collected in the United States and proportionately distributed between genders. The age range was between 21 and 82 years old with an average age of 56

### Sample collection and handling

Human plasma samples were obtained from five blood banks: Asterand, BioReclamation, BioSource, Geneticist, and Proteogenex. All cancer samples were confirmed by histology. All samples were collected through an IRB approved protocol (e.g., Protocol #AST-FPB-003, Western IRB) or a signed Waiver of Consent form. Individuals under the age of 18 or those who cannot consent for themselves were not included in the study. Samples were collected in the United States between 2013 and 2015.

Clinical information such as age, gender, pathology and stage, race, origin, smoking status, and sample collection dates were obtained. Whole blood samples were collected in EDTA tubes and stored at − 80 °C according to the biobank’s protocol. Plasma samples were transported on dry ice overnight to our sample storage site in Michigan City, Indiana, USA. Vials were inspected visually for damage upon receipt and stored at − 80 °C until analysis.

### Multiplexed immunoassay procedure

This study used a custom-made multiplexed immunoassay to measure the concentration of 21 biomarkers in human plasma samples. Sample collection and handling, and immunoassay procedure are consistent with our previous study (1, Supplementary Figure 1). Sample processing was performed by Eve Technologies Corporation (Calgary, Alberta, Canada). This assay reagent and format was validated against the 33-biomarker reagent used in the previous study [11] to ensure that all biomarkers performed similarly and maintained its congruity with the algorithm.

### Algorithm and statistical analysis

The algorithm considers duplicate measurements of the biomarkers from a patient and classifies each measurement as having NSCLC or not having NSCLC. If any of the measurement is classified as being from a subject with NSCLC, the subject is classified as having NSCLC. Since the implicit costs of allowing the disease to progress without treatment is greater than the cost of a false negative, the LCDT1 algorithm errs on the side of predicting that a subject has NSCLC.

A 5-PL curve was used to acquire the calibration curve. Data was cleaned based on preset criteria of ±20% coefficient of variation and removal of extrapolated and out of range data. Median, rather than average, was used to represent the central tendency of the plasma concentrations due to the skewed distributions and outliers. Normalization of diseased cohorts to healthy cohorts was examined for pattern recognition. *P*-values were calculated using *T*-tests, adjusted using Benjamini-Hochberg’s method for multiple comparisons [26]. The AUC was calculated for each biomarker and as a combined set of biomarkers. The ROC curve was used illustrate the performance of the model. Excel and R Version 3.4.4 were used for data analysis.

## Results

### Training set for optimizing the LCDT1 algorithm

In this study, we included the 33-biomarker model to examine congruity in using a higher set of biomarkers versus a smaller subset. Table 3 illustrates the algorithm performance using 33 versus 21 biomarkers are analogous. The LCDT1 algorithm was developed with slight modifications using a smaller subset of biomarkers from the 21. This information is proprietary and a patent application was filed. Patterns of up and down regulation of biomarkers were similar to our previous study [11]. The median concentration in LC patients compared to healthy non-smokers, asthma sufferers, and smokers was more than 200% higher in SAA (771%), MMP-9 (743%), IL-8 (535%), CXCL9/MIG (482%), TNFRI (406%), Gro (331%), MPO (300%), Rantes (274%), Resistin (271%), TNFRII (266), and MIF (219%). IL-2 and IL-7 showed greater than a 50% reduction in signal (Table 5).

**Table 3:** Results of Algorithm Models. Results of Optimized Algorithm Models (Training Set)

Biomarker	Algorithm 33	Algorithm 21	LCDT1 Algorithm
SE (95% CI)	92.8% (87.9, 96.1%)	97.4% (92.0, 99.5%)	92.4% (89.2, 94.3%)
SP (95% CI)	97.2% (95.5, 98.8%)	98.3% (95.4, 99.5%)	96.9% (95.2, 98.0%)

The LCDT1 algorithm was developed with slight modifications using a smaller subset of biomarkers from the 21. This information is proprietary and a patent application was filed

### Validation set performance

**Fig. 1:**
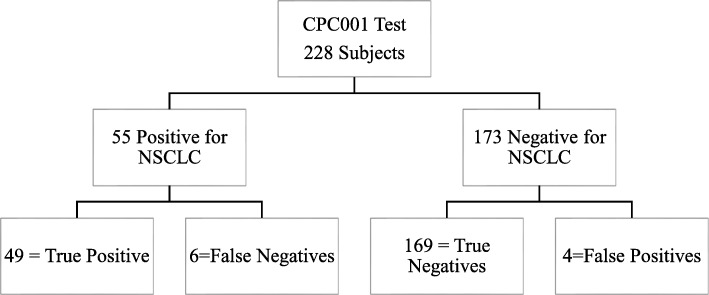
Validation Test Result

A novel blinded sample set of 228 (*N* = 456) subjects were processed in duplicate using the LCDT1. Of 228 subjects, 55 were Stage I NSCLC samples (Table 2). Our proprietary algorithm accurately detected 49 of the 55 Stage I LC samples (Fig. 1). There were 6 positive samples that were not detected and 4 negative LC samples that showed up as positive. The 4 samples that were false positives consisted of 3 breast cancers and 1 asthma sufferer (Supplementary Table 1). We were unable to follow-up with the patients to confirm if the breast cancer had metastasized into the lungs [27] or whether the asthma diagnosis was erroneously reached for an individual actually suffering of LC [28].

Algorithm 33 and the LCDT1 exhibit a similar accuracy rate of 95.6%, sensitivity of 89.1%, and a specificity of 97.7% in the validation test (Table 4). When only NSCLC cancers were analyzed, the specificity of both algorithms improved to 99.1%. This validation shows that the results are comparable using the 33 markers (from the previous study) versus the 21 or the LCDT1 markers (Table 4). Additional biomarkers were unnecessary to achieve the same clinical performance.

**Table 4:** Blind Test Performance for the 33, 21, and LCDT1 Algorithm (Validation Set)

	*Model*		
Statistics	Algorithm 33	Algorithm 21	LCDT1 Algorithm
Accuracy	95.6% (92.4, 97.7%)	94.3% (90.7, 96.8%)	95.6% (92.4, 97.7%)
Sensitivity	89.1% (78.9, 95.3%)	89.1% (78.9, 95.3%)	89.1% (78.9, 95.3%)
Specificity	97.7% (94.6, 99.2%)	96.0% (92.2–98.2%)	97.7% (94.6, 99.2%)
Positive Predictive Value (PPV)	92.5% (83.0, 97.4%)	87.5% (77.0, 94.2%)	92.5% (83.0, 97.4%)
Negative Predictive Value (NPV)	96.6% (93.1, 98.6%)	96.5% (93.0, 98.5%)	96.6% (93.1, 98.6%)
NSCLC Prevalence	24.1%	24.1%	24.1%
True Positive (TP)	49	44	49
True Negative (TN)	169	166	169
False Positive (FP)	4	6	4
False Negative (FN)	6	7	6

All entries show the statistical (95% CI). *Other cancer types were included in the analysis. Each subject consisted of two replicates (*N* = 2) or two data points processed by the algorithm. If one data point was positive, then the subject was considered positive for LC. Table was generated using R Version 3.4.4

### ROC curves and *P*-values

**Fig. 2:**
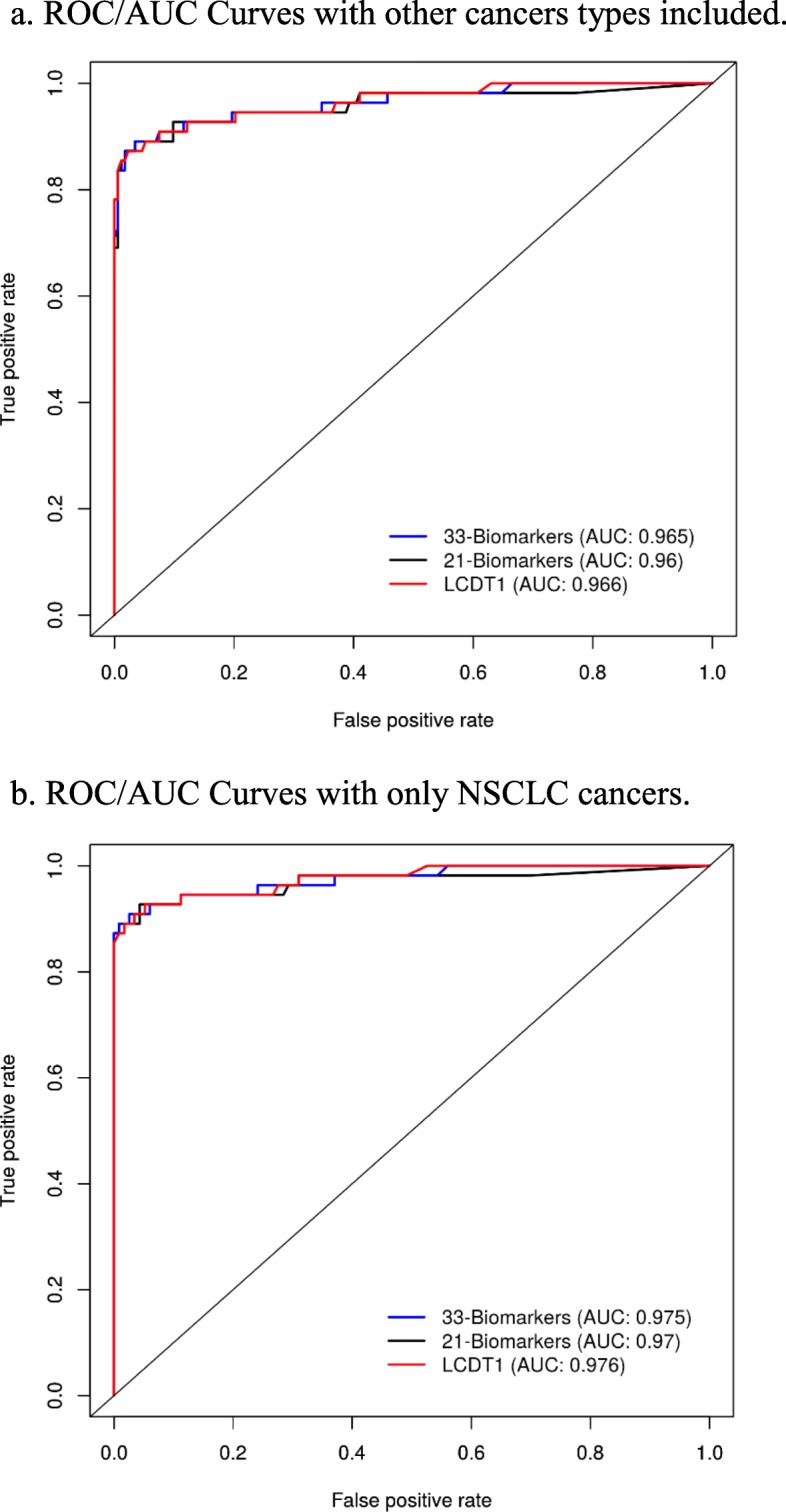
ROC/AUC Curves. a. ROC/AUC Curves with other cancers types included. b. ROC/AUC Curves with only NSCLC cancers. Figures were generated using R version 3.4.4

The Area under the ROC Curve (AUC) is the probability that an observation with a higher probability of being positive is positive. In our model, a ‘positive’ means that the model predicts that the subject has NSCLC. Although the discriminatory power, using AUC, for each individual biomarker was examined, it was not the determining factor in our selection process. The ROC/AUC for Algorithm 33, Algorithm 21, and the LCDT1 are 0.965, 0.960, and 0.966, respectively (Fig. 2a). When only NSCLC cancers were analyzed, the AUC for each algorithm improved by 0.01 (Fig. 2b). Once more, the *P*-values (*p* <  0.05) imply that several biomarkers are able to discriminate NSCLC from other pathologies to a degree (Table 5). These results (e.g., patterns, ROC/AUC, performance) provide a strong foundation for developing a clinical diagnostic test for NSCLC.

**Table 5:** Median Biomarker Concentrations and *P*-Value Using the Training Set

Biomarker	NSCLC Median [Q1-Q3], pg/mL	Asthma, Smokers, Non-Smokers Median [Q1-Q3], pg/mL	*P*-Value
CA125	26.4 [13.7–41.7]	13.6 [6.9–36.7]	0.073
CEA	2884.4 [1815.9–5573.8]	2115.3 [1194.8–3242.2]	0.003
CXCL9-MIG	4378.2 [2604.0–6844.5]	908.0 [539.0–1965.8]	< 0.001
CYFRA-21-1	5354.8 [3429.5–8090.4]	5088.3 [2939.9–9770.1]	0.026
GRO	2890.0- [2076.9–4178.0]	874.4 [507.5–1790.2]	< 0.001
HGF	869.4 [643.9–1647.1]	476.3 [271.9–1177.1]	0.006
IL-10	22.8 [11.7–38.2]	23.8 [14.0–45.8]	0.525
IL-12p70	21.1 [15.5–27.0]	19.9 [16.5–127.4]	0.082
IL-16	693.6 [345.1–1458.7]	717.5 [298.8–1469.4]	0.902
IL-2	11.5 [10.9–16.6]	33.8 [19.7–52.1]	0.005
IL-4	41.7 [25.3–51.6]	33.3 [22.5–50.3]	0.902
IL-5	17.9 [15.7–23.5]	28.7 [12.8–46.8]	0.188
IL-7	10.6 [10.6–10.6]	34.9 [18.9–61.1]	NA
IL-8	126.3 [44.5–323.8]	23.6 [15.9–42.3]	< 0.001
IL-9	11.9 [11.0–20.7]	22.3 [15.2–42.6]	0.016
Leptin	30,408.0 [16,682.6–45,886.3]	22,190.7 [8684.4–54,863.7]	0.224
LIF	45.5 [30.5–79.3]	39.6 [27.1–82.3]	0.511
MCP-1	530.2 [391.363–721.512]	372.8 [279.7–462.0]	< 0.001
MIF	865.6 [453.6–1501.3]	395.0 [196.7–1274.8]	0.752
MMP-7	1978.3 [1184.2–3190.33]	3585.2 [2671.9–5080.6]	< 0.001
MMP-9	93,587.2 [62,827.2–124,300.6]	12,593.8 [8856.8–19,799.6]	< 0.001
MPO	353,987.8 [246,376.2–616,739.2]	117,658.8 [69,768.5–212,726.3]	< 0.001
NSE	7273.5 [3852.3–10,487.8]	6576.1 [3806.6–46,981.4]	< 0.001
PDGF AB/BB	25,169.6 [21,611.8–30,055.0]	41,800.6 [26,115.3–53,016.0]	< 0.001
RANTES	105,356.2 [79,497.9–155,040.2]	38,458.4 [23,423.8–112,641.5]	0.003
Resistin	35,145.6 [25,185.8–53,466.7]	12,966.2 [9521.2–17,533.1]	< 0.001
SAA	6.55e7 [2.52e7–1.2e8]	8.5e6 [4.175e6–1.9825e7]	< 0.001
sCD40L	381.8 [155.9–752.5]	219.9 [110.285–628.7]	0.018
sEGFR	654.9 [544.0–1175.5]	936.5 [543.2–1943.2]	< 0.001
sFasL	229.8 [78.2–498.2]	263.7 [135.9–573.4]	0.03
sICAM-1	150,304.6 [123,699.9–187,843.8]	145,329.4 [117,164.7–182,796.7]	0.519
sTNFRII	15,477.5 [11,712.9–20,103.6]	5818.1 [4574.8–7295.3]	< 0.001
TNFRI	2514.8 [1748.7–3743.5]	619.5 [413.9–860.2]	< 0.001

Table was generated using R Version 3.4.4

## Discussion

Protein biomarkers have been extensively examined for diagnostic, prognostic, and therapeutic assessment of diseases and its treatments. Yet, many lab-developed assays never fully mature to penetrate the clinical setting [29]. Apart from the regulatory hurdles, there are many factors, such as sample collection, reagent manufacturing, and the acquisition of data, that may cause variability of end-results, which affects robustness and consistency, ~a requisite of any biological test used for clinical utility [30, 31]. Reducing the number of biomarkers was an important component of the present study as decreases complexity and the number of interactions between the antibodies, simplifies reagent production, and is more cost-effective [32].

In narrowing our list, the biological justification for the selection of biomarkers was critical in avoiding numerical quirks that may mask the true driver of a physiological process [11]. To elaborate, the statistical model in the previous study was a Random Forest (RF) model. When an RF model is fit, a measure of the variable’s importance is calculated. In this case, the variables are the biomarkers. The variable’s importance is defined as how well, on average, the biomarker increases the distinction of groups in the model (in our case NSCLC and not-NSCLC). Here, the Node impurity (how well the trees partition the data at each step in the algorithm) is measured using the Gini index [33].

Due to the naturally occurring relationships between the biomarkers examined, depending on variable’s importance as the sole factor in determining if a biomarker should stay in the smaller set of biomarkers to develop the new model, is not viable. If any two biomarkers are highly correlated, then the ‘importance’ of one biomarker is masked by the other biomarker. This is because both biomarkers would provide the same information to the model thereby making the excluded biomarker redundant. Therefore the ‘redundant’ biomarker, seeming insignificant, could have served as a substitute for the included biomarker [34].

However, if the two biomarkers are statistically correlated, but only one is biologically related to the disease, we may not be able to determine which biomarker is truly more important to the underlying biological mechanisms. Thus, biological relevance and patterns weighed heavily.

Many of the markers in our set have been studied for decades and have been shown to have potential for diagnosing LC [35–39]. In our studies, certain biomarkers were elevated at higher levels or depressed depending on whether we were looking at early stage (I-II) or late stage (III-IV) NSCLC patients, e.g., the upregulation of CEA and CYFRA-21-1 (common cancer markers widely studied) [36] were not as prominent in early stage NSCLC. The occurrence of a lower expressed CYFRA in the early stages of NSCLC has been indicated by Guergova-Kuras M, et al. [37] using monoclonal antibodies to detect early stage NSCLC. This phenomenon of varying marker levels at different stages of NSCLC is not surprising as protein abundance reflects current physiological state of the disease.

Examples of the markers that were elevated in stage I-II NSCLC were IL-8, MMP-9, and SAA. The synergistic regulation and pathways of these markers correlates with previous scientific findings: For example, IL-8 is a multi-functional chemokine that induces chemotaxis and phagocytosis, promotes angiogenesis, and aids in maintenance of mesenchymal features in carcinoma cells [40, 41]. Robust upregulation of CXCL8 (aka IL-8) has been observed in erlotinib-resistant cell lines [41] which also makes it a cancer therapy target. A study by Liu et al. using 141 NSCLC patients indicated that IL-8 may have up-regulated MMP-9 in lymph node metastasis of NSCLC patients [38].

MMP-9 is a widely studied protease that cleaves extracellular matrix (ECM) proteins to regulate ECM remodeling [42]. MMP-9 is involved in basement membrane degradation that furthers tumor invasion and metastases [42]. Past studies showed that MMP-9 s are highly elevated in LC patients, especially stage III-IV [43, 44]. We also observed a correlation between IL-8 and MMP-9 levels in LC patients.

SAA is an apolipoprotein that is secreted during acute phase inflammation and is a known LC biomarker. Sung et al. measured 180 healthy and 170 lung adenocarcinoma plasma or serum samples and found a 14-fold increase of SAA levels in the LC patient [45]. Another by Biaoxue, R. et al. indicated that SAA alone could detect LC with 0.59 sensitivity and 0.92 specificity [39]. We measured a six-fold increase in SAA levels at all stages of NSCLC compared to healthy controls.

Proteins such as IL-8, MMP-9, and SAA are involved in physiological inflammatory processes. Some of these proteins are highly expressed in specific cancers, while others are inhibited. Independently, each protein has the ability of discriminating healthy from disease patients. When LC biomarkers are multiplexed and combined with an algorithm and additional demographic data, its diagnostic capability increases and could serve as a powerful clinical tool.

Using biomarkers for diagnosing diseases requires constant revalidation to ensure that it remains applicable to the intended population. Like any method, biomarkers have limitations as they are affected by sample origin, ethnicity, gender, environmental and carcinogenic exposure, and reagent and platform variations. Strict quality assurance and processes from the bench (e.g., developing reagents) to the clinic (e.g., collecting samples) to the acquisition of the end result (e.g., data cleaning and processing) are imperative. Furthermore, statistical and machine learning algorithms also need to be tested for bias and refined as new data are collected.

Despite, these limitations, biomarkers in conjunction with machine learning methods serve as an important component in fighting cancer as they provide benefits. Such advantages include a means of a simple, non-invasive method in detecting cancer; acquiring prognostic information, and assessment of the efficacy of therapeutic methods.

## Conclusions

We aimed to develop an accurate test that was specific to early stage NSCLC. A multi-cancer test, though remarkable, could increase patient anxiety and fiscal expense due to additional (possibly unnecessary) follow-up procedures. These concerns are mirrored in medical practitioners’ reluctance to order full body imaging in asymptomatic patients [46].

This study shows that we were able to successfully reduce the number of biomarkers from 33 to 21, while maintaining a high performance in detecting early stage NSCLC. The LCDT1 is 97.7% specific for Stage I NSCLC even when other cancer types were present. An estimated 9 out of 10 (89.1% sensitive) early stage LC patients would be detected by the LCDT1. The LCDT1 is 95.6% accurate.

As a diagnostic test, physicians prefer tests with high sensitivity and sacrifice specificity. The argument is that not detecting “a” cancer is more detrimental than a false negative. A highly sensitive diagnostic test is important where the test is used to identify a serious but treatable disease; and a highly specific test avoids further subjection of the patient to unnecessary follow-up medical procedures. In the case of LC, current diagnostic methods (i.e., CT, PET) have high sensitivity but low specificity. If patients who are suspected to have a lung nodule on a CT are given a second test with a low (or high) sensitivity and high specificity, then nearly all of the false positives could be identified as disease free.

Our clinical goal is to decrease risks and unnecessary procedures to patients without delaying curative treatment [47] and increase access to communities with social and economic barriers. The LCDT1 is a simple blood test with great potential for clinical applications in detecting Stage I NSCLC. When used with gold standards such as the CT/PET scans in conjunction with algorithms and improved PN guidelines, could mean a significant reduction in the number of false negatives and an increase in early stage detection.

## Supplementary information

**Additional file 1.** Supplementary Figure 1. Immunoassay Procedure.

**Additional file 2.** Supplementary Table 1. Actual and predicted results using the LCDT1 Algorithm.

## Data Availability

The data analysed during the current study are available from the corresponding author upon reasonable request and with permission of Lung Cancer Proteomics. Due to proprietary rights, restrictions may apply to the availability of these data and so are not publicly available. Datasets, including supplementary information, during this study are included in this published article and from a previous study as noted on the article.

## Abbreviations

CT: Computed tomography
FNR: False negative rate
FPR: False positive rate
LC: Lung cancer
LCDT1: Lung cancer detector test 1
LDCT: Low-dose spiral computed tomography
NSCLC: Non-small cell lung cancer
PET: Positron emission tomography
PN: Pulmonary nodules
USPSTF: US Preventive Service Task Force
