# Supplementary material for: Blood test shows high accuracy in detecting stage I non-small cell lung cancer
Source: BMC Cancer. 2020 Feb 21;20:137. doi: 10.1186/s12885-020-6625-x (PMC7035746; doi:10.1186/s12885-020-6625-x)
Supplement: Supplementary file 1 — Additional file 1. Supplementary Figure 1. Immunoassay Procedure. [file 12885_2020_6625_MOESM1_ESM.docx]

#### Supplementary Figure 1. Immunoassay Procedure.

| 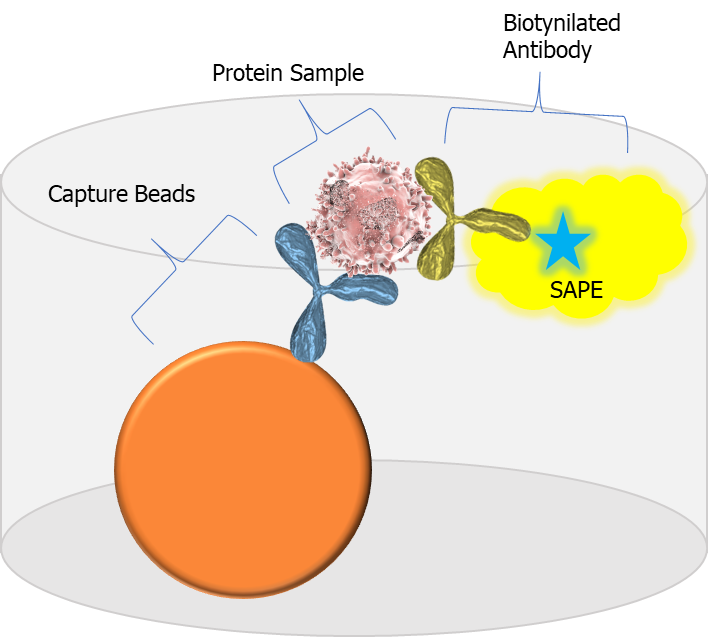 | **Step 1:** Incubate sample with conjugated beads  **Step 2:** Incubate with biotinylated antibody  **Step 3:** Incubate with SAPE  **Step 4:** Read fluorescence  The interaction between the plasma sample, beads and antibodies occur in one well. |
| --- | --- |

The reagent kits were designed on magnetic beads using a capture sandwich immunoassay format. Human plasma samples were incubated overnight (16-18 hours) at 2-8°C with the capture antibody–coupled beads. The next day, the beads were washed 2 times and incubated with the detection antibodies for one hour. All washes and reagent transfers were performed using a semi-automated process by ViaFlo96 from Integra Biosciences (Hudson, NH, USA). Second day incubations were also performed at room temperature (20-25°C) at 500-800 rpm. A reporter Streptavidin-Phycoerythrin conjugate (SA-PE) was added and incubated for 30 minutes. The excess detection antibody and SA-PE beads are removed using 2 washes. Sheath fluid is then added to the beads, and the plate is placed on the shaker for 5 minutes. The plates were read using the Flex Map 3D (Luminex Technologies, Austin, TX, CA). The Bio-Plex Manager 6.1 (Bio-Rad Laboratories, Hercules, CA, USA) was used for data acquisition using a 5PL logistic curve to obtain analyte concentrations.
