# Supplementary material for: Blood test shows high accuracy in detecting stage I non-small cell lung cancer
Source: BMC Cancer. 2020 Feb 21;20:137. doi: 10.1186/s12885-020-6625-x (PMC7035746; doi:10.1186/s12885-020-6625-x)
Supplement: Supplementary file 2 — Additional file 2. Supplementary Table 1. Actual and predicted results using the LCDT1 Algorithm. [file 12885_2020_6625_MOESM2_ESM.docx]

#### Supplementary Table 1. Actual and predicted results using the LCDT1 Algorithm.

|  |  | ***Predicted*** | |  |
| --- | --- | --- | --- | --- |
|  |  | **Not-NSCLC** | **NSCLC** | **Total Actual** |
|  | **Asthma** | 10 | 1 | 11 |
|  | **Breast** | 37 | 3 | 40 |
|  | **CRC** | 5 | 0 | 5 |
| **Actual** | **Non-Smoker** | 57 | 0 | 57 |
|  | **NSCLC** | 6 | 49 | 55 |
|  | **Pancreatic** | 3 | 0 | 3 |
|  | **Prostate** | 9 | 0 | 9 |
|  | **Smoker** | 48 | 0 | 48 |
|  | **Total** | 175 | 53 | 228 |

*Table was generated using R Version 3.4.4.
